# VOICES IN CLIMATE ACTION: INTERGENERATIONAL PERSPECTIVES AMONG OLDER ADULT ENVIRONMENTAL ACTIVISTS

**DOI:** 10.1093/geroni/igae098.0860

**Published:** 2024-12-31

**Authors:** Zeyu Liu, Marie Cope, Leslie Schultz, Karl Pillemer

**Affiliations:** Cornell University, Jersey City, New Jersey, United States; Cornell University, Ithaca, New York, United States; Cornell University, Ithaca, New York, United States; Cornell University, Ithaca, New York, United States

## Abstract

Intergenerational collaboration is a critical approach for addressing climate change and related environmental challenges. However, research has also uncovered underlying intergenerational tensions and ageism in public discourse around climate change. A gap in existing research is the perspectives of older adults on the role of younger people’s involvement in and intergenerational connections relating to climate change action. This study inductively analyzed data using a thematic analysis approach to identify and categorize perceived intergenerational issues in climate change. We conducted semi-structured interviews with 45 committed climate change activists (10+ hours a week of engagement), ages 65-89. The analysis revealed four major intergenerational themes: 1) concern for future generations as a motivator for climate change action; 2) benefits of intergenerational engagement around climate change; 3) barriers to intergenerational interaction/collaboration on climate change; 4) opportunities for mutual mentoring. Generativity emerged as a major overarching theme, in which respondents expressed both a sense of responsibility for the current situation and a desire to help and inspire younger people. The findings emphasize the importance of shared environmental concerns and active participation in facilitating communication between older and younger individuals. The study also identified challenges such as role transitions and differing perspectives on environmental responsibility. Based on these insights, recommendations are provided to enhance the effectiveness of intergenerational environmental programs, highlighting the need for strategic interaction management and the integration of diverse generational viewpoints.

